# Implementation of integrated stepped care for unhealthy alcohol use in HIV clinics

**DOI:** 10.1186/s13722-015-0048-z

**Published:** 2016-01-13

**Authors:** E. Jennifer Edelman, Nathan B. Hansen, Christopher J. Cutter, Cheryl Danton, Lynn E. Fiellin, Patrick G. O’Connor, Emily C. Williams, Stephen A. Maisto, Kendall J. Bryant, David A. Fiellin

**Affiliations:** Yale University School of Medicine, 367 Cedar Street, ESH A, New Haven, CT 06510 USA; Center for Interdisciplinary Research On AIDS, Yale University School of Public Health, 135 College Street, New Haven, CT 06510 USA; College of Public Health, University of Georgia, 131 Wright Hall, Health Sciences Campus, Athens, GA 30602 USA; VA Puget Sound Health Care System, Center of Innovation for Veteran-Centered and Value-Driven Care, 1100 Olive Way, Suite 1400, Seattle, WA 98101 USA; Department of Health Services, University of Washington, 1959 NE Pacific Street, Magnuson Health Sciences Center, Room H-664, Seattle, WA 98195 USA; Syracuse University, 430 Huntington Hall, Syracuse, NY 13244 USA; National Institute on Alcohol Abuse and Alcoholism HIV/AIDS Program, 5635 Fishers Lane, Bethesda, MD 20892-7003 USA

**Keywords:** HIV, Alcohol-related disorders, Qualitative methods, Diffusion of innovation

## Abstract

**Background:**

Effective counseling and pharmacotherapy for unhealthy alcohol use are rarely provided in HIV treatment settings to patients. Our goal was to describe factors influencing implementation of a stepped care model to address unhealthy alcohol use in HIV clinics from the perspectives of social workers, psychologists and addiction psychiatrists.

**Methods:**

We conducted two focus groups with Social Workers (n = 4), Psychologists (n = 2), and Addiction Psychiatrists (n = 4) involved in an ongoing randomized controlled trial evaluating the effectiveness of integrated stepped care for unhealthy alcohol use in HIV-infected patients at five Veterans Health Administration (VA) HIV clinics. Data collection and analyses were guided by the Consolidated Framework for Implementation Research (CFIR) domains, with a focus on the three domains which we considered to be most relevant: *intervention characteristics* (i.e. motivational interviewing, pharmacotherapy), the *inner setting* (i.e. HIV clinics), and *characteristics of individuals* (i.e. the providers). A multidisciplinary team used directed content analysis to identify major themes.

**Results:**

From the providers’ perspective, the major implementation themes that emerged by CFIR domain included: (1) *Intervention characteristics*: providers valued tools and processes for facilitating patient motivation for treatment of unhealthy alcohol use given their perceived lack of motivation, but expressed a desire for greater flexibility; (2) *Inner setting*: treating unhealthy alcohol use in HIV clinics was perceived by providers to be consistent with VA priorities; and (3) *Characteristics of individuals:* there was high self-efficacy to conduct the intervention, an expressed need for more consistent utilization to maintain skills, and consideration of alternative models for delivering the components of the intervention.

**Conclusions:**

Use of the CFIR framework reveals that implementation of integrated stepped care for unhealthy alcohol use in HIV clinics is facilitated by tools to help providers enhance patient motivation or address unhealthy alcohol use among patients perceived to be unmotivated. Implementation may be facilitated by its consistency with organizational values and existing models of care and attention to optimizing provider self-efficacy and roles (i.e. approaches to treatment integration).

## Background

Unhealthy alcohol use, defined as the spectrum of drinking including at-risk drinking and alcohol use disorders [1], is common among HIV-infected patients and associated with a range of adverse health outcomes. Unhealthy alcohol use among HIV-infected patients is associated with poor antiretroviral therapy (ART) adherence [2–8]; impaired treatment response; and decreased quality of HIV care [5, 6, 9]. In turn, individuals who stop drinking experience an improved response to HIV therapy [5, 10]. Further, unhealthy alcohol use increases the risk of important medical comorbidites, such as cardiovascular disease [11], liver disease [12] and malignancy [13] and is associated with sexual risk behaviors and ongoing HIV transmission [14, 15].

### Integration of treatment for unhealthy alcohol use into HIV clinics

Despite the high prevalence and adverse effects of unhealthy alcohol use, few effective interventions for unhealthy alcohol use are offered in HIV clinics [16–21]. Interventions that have been shown to be effective for decreasing unhealthy alcohol use in uninfected patients, including brief interventions for at-risk drinking [22], Motivational Enhancement Therapy, and alcohol pharmacotherapies [23, 24] are not routinely provided in HIV clinics [25, 26]. One advantage of integrating the treatment of unhealthy alcohol use into HIV clinics is that it provides “one stop shopping” and limits potential losses to follow-up seen with referral to off-site treatment. However, integrated treatments are often based on screening strategies and must contend with low levels of knowledge regarding alcohol-associated health risks and motivation among non-treatment seeking individuals [27, 28]. Also, providers often neglect to discuss or recognize unhealthy alcohol use [29–32] and are underprepared to provide treatment [33]. In addition, treatments for unhealthy alcohol use are not uniformly effective, which provides a rationale for stepped care approaches. Stepped care approaches, which have been applied to chronic disease conditions (e.g. depression and hypertension), typically start with a low-intensity evidence-based intervention with increasing the intensity of treatment if patients fail to respond to initial treatment strategies [34, 35].

### The innovation: integrated stepped care for unhealthy alcohol use in HIV clinics

To address a need for effective treatment models for unhealthy alcohol use among HIV-infected patients through HIV clinics, we are conducting the *Starting Ethanol Treatment in Primary care Trial (STEP Trials)*, a series of three linked multi-site randomized clinical trials designed to evaluate the implementation and effectiveness of integrated stepped care among HIV-infected patients with unhealthy alcohol use. Briefly, HIV-infected patients are recruited into one of three trials based on their alcohol use—(1) at-risk drinking [36], (2) moderate alcohol consumption in the presence of liver disease (MALD) (defined as any alcohol consumption in the past month in the presence of a FIB-4 score greater than 1.45, a validated marker of liver fibrosis [12, 37, 38], or active Hepatitis C virus infection based on serology and presence of a detectable viral load) or (3) alcohol abuse or dependence criteria [39]. Each trial compares integrated stepped care to treatment as usual.

Patients are recruited from one of five Infectious Disease Clinics within the Veterans Health Administration (VA). Patients in the stepped care arm receive evidence-based interventions of increasing intensity (or not) based on their response to initial treatments. Social workers deliver a brief intervention (the Brief Negotiated Interview [BNI]) [40–42] with a two-week telephone booster to patients with at-risk drinking or MALD; Psychologists deliver four sessions of Motivational Enhancement Therapy [43]; and Addiction Psychiatrists provide Addiction Physician Management, with an emphasis on offering pharmacotherapy. Addiction Psychiatrists were encouraged to prescribe pharmacotherapy based on what they thought was most likely to benefit the individual patient. This includes Food and Drug Administration (FDA) approved pharmacotherapies for alcohol dependence (i.e. disulfiram, naltrexone, acamprosate) and prescribing of medications with evidence of efficacy (e.g. topiramate, gabapentin).

To enhance implementation and consistency of the intervention across sites, providers received an initial training and are provided with manuals and structured encountered forms to guide each visit. Providers are also given a “Feedback Form” to review patient-specific data, including self-reported alcohol use, other substance use, level of bother by alcohol use, motivation for treatment, and biomarkers known to be impacted by alcohol [liver function, HCV status, HIV biomarkers, and VACS Index score (a validated prognostic marker of morbidity and mortality found to be responsive to levels of alcohol use)] [44–46] with the patient. Visits with providers occur within the HIV Clinic or nearby available office-space. The *STEP Trials* is occurring in the context of current VA initiatives focused on developing patient-centered medical homes, known as Patient Aligned Care Teams (PACTs), and its extensions (i.e. Primary Care-Mental Health Integration [PC-MHI] and Health Promotion and Disease Prevention [HPDP] Programs) [47, 48]. These VA programs have been developed to promote the use of multidisciplinary teams to address a range of health related issues in the context of co-located and collaborative care within primary care settings [48].

Consistent with effectiveness-implementation hybrid study designs intended to evaluate a clinical intervention while collecting data on implementation [49], we conducted the current qualitative study to examine perspectives of providers on the implementation of integrated stepped care for unhealthy alcohol use and inform implementation efforts in other HIV clinical settings. Given its suitability for examining factors impacting implementation of clinical interventions across different settings and disciplines, including addiction treatment, we used the Consolidated Framework for Implementation Research (CFIR) to guide data collection and a directed content analysis [50–54].

## Methods

### Study overview

**Fig. 1:**
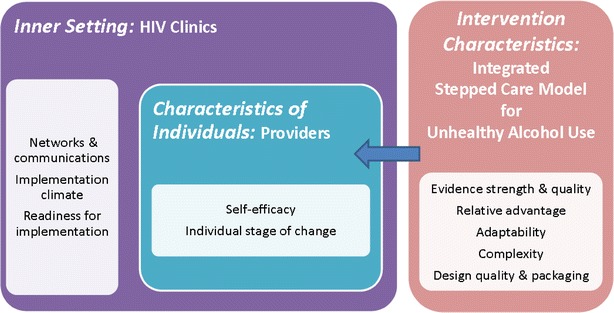
Integrated stepped care for unhealthy alcohol use in HIV clinics: relevant CFIR domains and constructs*. *Adapted from Damschroder LJ, Aron DC, Keith RE, Kirsh SR, Alexander JA, Lowery JC. Fostering implementation of health services research findings into practice: A consolidated framework for advancing implementation science. CFIR Figure and Explanatory Text (http://www.implementationscience.com/content/supplementary/1748-5908-4-50-s1.pdf)

After approximately 1 year of project implementation and patient care, we conducted two focus groups with Social Workers, Psychologists and Addiction Psychiatrists who were delivering stepped care to address unhealthy alcohol use in HIV clinics in the context of the *STEP Trials*. We chose qualitative methods given their appropriateness for studying complex processes with regards to patient, physician and organizational-level factors associated with implementation of evidence-based interventions [55]. We used a directed content analysis [56, 57] due to its successful use in the past to elucidate implementation efforts according to the CFIR and other implementation science constructs [58–61] (Fig. 1). The protocol was approved by Yale University’s Human Investigations Committee and VA Connecticut Healthcare System’s Institutional Review Board.

### Study design and participants

While the VA-based healthcare system is an integrated healthcare system, including both primary HIV care and addiction treatment services within the same medical center complex, the level of direct integration of providers into HIV clinics in the context of routine clinical care (i.e. not in the context of the *STEP Trials)* differs across sites (Table 1). We recruited members of the three types of providers (i.e. Social Workers, Psychologists, and Addiction Psychiatrists) from the five participating HIV clinics who were involved with the *STEP Trials*. The focus groups were conducted as part of a national meeting whereby participants attended based on availability. None of the potential interventionists declined to participate though some were unable due to their unavailability to travel to the meeting. We chose to conduct focus groups given our expectation that the group interaction would generate unique insights about shared experiences, while also allowing us to learn about differences across sites [55]. All providers gave verbal informed consent and the study was HIPAA compliant.

**Table 1:** Pre-implementation provider integration into HIV clinic and additional considerations by site

HIV clinic characteristics	Social worker	Psychologist	Addiction psychiatrist	Additional considerations
Site 1	Yes	No	No	n/a
Site 2	Yes	Yes	No	n/a
Site 3	No	Yes	No	n/a
Site 4	No	No	No	n/a
Site 5	No	No	No	Providers at geographically dispersed VA-based locations

### Data collection

The two focus groups were facilitated by investigators with experience conducting qualitative research [62–68]. One focus group (facilitated by EJE, DAF, NH and SM) consisted of Social Workers and Psychologists and the other (facilitated by EJE, DAF, LEF) consisted of Addiction Psychiatrists.

### Focus group procedures

Based on the CFIR framework [54] and multi-disciplinary input, we used grand tour questions (i.e. open ended questions intended to stimulate discussion), and probes (i.e. prompts) to examine the perceived impact of *intervention characteristics*, *inner setting* and *characteristics of the individuals* (Fig. 1) on the delivery of integrated stepped care for unhealthy alcohol use in HIV clinics (Text Box) [69]. These CFIR domains were chosen as the focus of this investigation as they were the most directly relevant to the experiences of the providers delivering this innovative intervention and consistent with prior guidance regarding how to apply the CFIR [54, 59].

Providers were encouraged to discuss their experience of delivering integrated stepped care in the context of their usual role and not as part of a research project, per se. They were instructed that their responses would be kept confidential, would not impact their current employment or any aspect of involvement in the research. They were also invited to complete a brief demographic survey after the focus groups were completed. The focus groups were recorded, professionally transcribed, and reviewed for accuracy [55].

**Table:** Text Box: Focus Group Guides: Example Grand Tour Questions with Probes

***Intervention Characteristics*** **: Can you tell me about your experiences with treating HIV-infected patients for their unhealthy alcohol use in the context of the** ***STEP Trials*** **?**
How does the integrated stepped care approach compare to other treatment approaches?
What made it easier?
What make it more difficult?
Does the intervention feel like something you would create? How would you change it?
***Intervention Characteristics:*** **How did you find the patients responded to your treatment and the integrated stepped care treatment approach, in general?**
How motivated were patients to participate?
What seemed to impact their motivation?
How could this have been improved?
***Characteristics of the Individuals:*** **What was your experience with delivering the intervention?**
What aspects did you find most effective?
What aspects did you find least effective?
How did it fit in with your other roles and responsibilities?
How comfortable are you in delivering this type of intervention?
What changes would you make in the training materials to increase their usability?
***Inner Setting:*** **Can you tell me more about how you communicated with others involved in the care of your patients with unhealthy alcohol use?**
What about social workers/psychologists/psychiatrists?
What about the patients’ primary care provider?
***Inner Setting:*** **How important is treating unhealthy alcohol use to your supervisor? To the VA?**
Does this get measured in any way?
What kinds of resources are dedicated to this?
Do you think your supervisor would support you to do this kind of work even after the trial ends?
Describe some of the complexities in implementing this treatment model with regards to the duration, scope, disruption of other activities, number of steps.

Grand tour questions in bold; CFIR constructs noted in bold with italics

### Qualitative analysis

Six individuals of our multidisciplinary team, including members not present at the focus groups and one member not previously involved in the project, applied content analysis with a deductive approach to code the transcripts [56, 57]. Team members were trained on the CFIR framework [54] in advance and used the domains and constructs as predetermined codes [52]. The transcripts were independently reviewed line by line, and elements of the CFIR domains and constructs were assigned to meaning units (i.e. transcript text) [57]. Team members met to discuss emerging ideas, resolve discrepancies and reach consensus regarding the codes. One author (EJE) then re-reviewed the transcripts to systematically apply these codes. To organize the data and facilitate retrieval, we used qualitative analysis software (AtlasTi version 6.2, Berlin, Germany). We organized the transcript text by domains and constructs and then re-reviewed the quotes for themes and illustrative text and discussed as a group. To increase the rigor of our approach, we reviewed a summary of our findings with participants to confirm that we had accurately understood and conveyed their experiences [55].

## Results

Participating providers included Social Workers (n = 4), Psychologists (n = 2) and Addiction Psychiatrists (n = 4) (Table 2). We present themes and representative quotes organized by the CFIR domains and their sub-domains, with a focus on *intervention characteristics*, the *inner setting*, and *characteristics of the individuals.* The definitions used for each domain and sub-domain as applied to the *STEP Trials* are listed in Table 3.

**Table 2:** Relevant CFIR Domains with definitions as applied to integrated stepped care for unhealthy alcohol use

Domain	Definition
*I. Intervention characteristics: integrated stepped care for unhealthy alcohol use*	
Evidence strength & quality	Providers’ perception of the quality and validity of evidence supporting the belief that integrated stepped care for unhealthy alcohol use will lead to decreased unhealthy alcohol use
Relative advantage	Providers’ perception of the advantage of implementing integrated stepped care for unhealthy alcohol use versus an alternative solution
Adaptability	The degree to which integrated stepped care for unhealthy alcohol use can be adapted, tailored, refined, or reinvented to meet local needs
Complexity	Perceived difficulty of implementation, reflected by duration, scope, radicalness, disruptiveness, centrality, and intricacy and number of steps required to implement
Design quality & packaging	Perceived excellence in how integrated stepped care for unhealthy alcohol use is bundled, presented, and assembled
*II. Inner domain: HIV clinics*	
Networks and communications	The nature and quality of webs of social networks and the nature and quality of formal and informal communications within VA-based HIV clinics and across providers
Implementation climate	The absorptive capacity for change, shared receptivity of involved individuals to integrated stepped care for unhealthy alcohol use and the extent to which use of it will be rewarded, supported, and expected within the VA
Tension for change	The degree to which providers perceive the current situation as intolerable or needing change
Compatibility	The degree of tangible fit between meaning and values attached to integrated stepped care for unhealthy alcohol use by involved individuals, how those align with individuals’ own norms, values, and perceived risks and needs, and how this treatment model with existing workflows and systems
Relative priority	Individuals’ shared perception of the importance of the implementation within the VA-based HIV clinics
Readiness for implementation	Tangible and immediate indicators of organizational commitment to its decision to implement integrated stepped care for unhealthy alcohol use
Available resources	The level of resources dedicated for implementation and on-going operations, including money, training, education, physical space, and time
Access to knowledge & information	Ease of access to digestible information and knowledge about the intervention and how to incorporate it into work tasks
*III. Characteristics of individuals: providers*	
Self-efficacy	Providers’ belief in their own capabilities to execute courses of action to achieve implementation goals
Individual stage of change	Characterization of the phase a provider is in, as he/she progresses toward skilled, enthusiastic and sustained use of integrated stepped care for unhealthy alcohol use

Adapted from Damschroder LJ, Aron DC, Keith RE, Kirsh SR, Alexander JA, Lowery JC. Fostering implementation of health services research findings into practice: A consolidated framework for advancing implementation science. CFIR Constructs with Short Definitions (http://www.implementationscience.com/content/supplementary/1748-5908-4-50-s3.pdf)

**Table 3:** Provider characteristics

Characteristic	Overall (n = 9)	Social workers (n = 4)	Psychologists (n = 2)	Addiction psychiatrists (n = 3)
Age, mean (SD) in years	44 (11)	35 (8)*	54 (1)	45 (11)
Race (%)				
White	78	100	50	67
Black	11	0	50	0
Asian	11	0	0	33
Ethnicity, Hispanic (%)	11	25	0	0
Gender, Female (%)	89	100	100	67
Average number weekly patients, mean (SD)	23 (20)	20 (18)	23 (18)	29 (29)
Number HIV-infected, mean (SD)	8 (11)	15 (14)	2 (1)	2 (1)
Number with an alcohol use disorder or alcohol related problem, mean (SD)	12 (12)	5 (5)	16 (20)	17 (14)
Years in current role, mean (SD), [range]	8 (8), [1–24]	5 (7), [1–15]	6 (4), [3–9]	14 (10)

One social worker did not provide data on age and focus groups included one additional Addiction Psychiatrist who declined to complete the survey; thus these data are missing

## Intervention Characteristics Domain: Integrated Stepped Care Model

Providers indicated that the integrated stepped care model quality, content and packaging facilitated implementation, yet believed it would benefit from adaptability with regards to content, duration and source of the intervention.

### Evidence strength and quality

Social Workers and Psychologists, alike, endorsed fundamental aspects of the intervention, such as motivational interviewing, and recognized that it was evidence-based and believed it to be of high quality. For example, one provider described:

*I come from an addiction background before coming to the Infectious Disease clinic, and my mental health background. So motivational interviewing is kind of like magic dust*$\ldots$

Similarly, the Addiction Psychiatrists were familiar with strong data supporting the use of pharmacotherapy to treat alcohol use disorders as recommended within the stepped care model:

*I was thinking about the COMBINE Study and the medical management was a very powerful intervention*$\ldots$*I recall from Project COMBINE that included recommendations of going to AA, but it also included me prescribing medication.*

### Relative advantage

As participants were often not treatment seeking, the providers found it helpful that the intervention contained tools, such as personalized normative feedback, for promoting motivation:

*The one where it talked about your drinking [compared to norms], in both my cases they were surprised by the results. It was palpable. You could tell. They*$\ldots$*you know. What do you think of what I’ve just said in terms of, this shows there is such*—*this high percentile [compared to] drinking [norms] the language is*–*was surprising to them.*

### Adaptability

Providers reported a desire for increased flexibility regarding the number of treatment sessions, as well as an ability to expand the scope of the focus to address other prevalent and important risk behaviors. On one hand, they felt that more time was needed to effectively build rapport and expressed a desire to be able to tailor the number of treatment sessions to participant needs. On the other hand, there was an expressed preference to maintain the treatment number as finite. For example, one Psychologist reported:

*The one thing I probably would change, I think I would want more than, maybe just a little more than four sessions, for me*$\ldots$*My guys worked, so we had to kind of shuffle around to get ‘em in on off days, so committing to 4 sessions was easy. But I felt like they*—*you were just at that point where you’re holding your kids’ hips as he’s bicycling without training wheels and I just let him go, and he was just taking off*—*Session number 4. Done. And if I only had just a 5th, maybe just a 5th session, would be the one thing that I would change*$\ldots$*[But] I’m all for*$\ldots$*therapy ending.*

Similarly, one Addiction Psychiatrist echoed these sentiments:

$\ldots$*you start to build a [rapport], and then, because of this session slots issue you cannot see him anymore.*

Providers were also eager to extend the intervention to address drug use in addition to alcohol use:

*The only other thing too that I also thought about in terms of I think all of our patients, we’re only focusing on alcohol use*$\ldots$*we have like a huge crack epidemic too*$\ldots$*So it’s also interesting to me that we’re only talking to them about how alcohol impacts their body, and I’m ignoring the fact*$\ldots$*he’s like, “No, I did not drink, I just smoked crack yesterday.” Great. You know.*

In addition, there was a stated interest by one Social Worker to extend the focus from impact on health to HIV prevention efforts:

*I mean, talking about the relationship between your sex practices and your substance use, talking about the relationship between lack of condom use and substance use, that’s all huge in our clinic. There’s a huge correlation between that. Even to asking them about, are you disclosing your sex*—*when they go to sex parties, there’s a whole, that’s a whole ‘nother*-$\ldots$*I would totally be interested in seeing how we could do best practices in the VA with prevention positives.*

### Complexity

Some aspects of integrated stepped care for unhealthy alcohol use were perceived to be complex. Identifying appropriate individuals for the intervention was among the greatest challenges. Providers expressed a need for clear processes for identifying HIV-infected individuals with unhealthy alcohol use:

*Yeah, unless you had, when they come to the clinic, you provide them with a packet of paperwork for them to fill out, and you have the AUDIT*-*C or whatever other screening tools you’re using, I think it’s going to be difficult to implement.*

Other challenges with implementation of the intervention included the time involved as well as management of the logistics. It was felt that dedicated staffing was needed to manage the logistics involved with intervention implementation. According to one Social Worker:

*Somebody would have to be tracking to make sure that it went the right way. There’s gotta be a way*$\ldots$*in terms of tracking someone would have to be responsible for it. It might be unrealistic to think that with 1100 or 1500 person caseloads, a Social Worker would really be able to do that well. I mean, things would fall through the cracks.*

With regards to the time involved in delivering the intervention, one Social Worker reflected on the complexity of implementing the intervention with regards to time and logistics:

*I think the word “brief” can be deceiving sometimes*$\ldots$*I don’t think that 20* *min which I think was the original design was always necessarily [enough time]*- *I think sometimes it drags out more so to 40 something to really bring ‘em around. And then the phone booster depending on, same thing, like the resource needs, can be a challenge. And in my real world I think scheduling a phone appointment, if it’s not [the research coordinator] reminding me that I have to do the phone appointment, and me finding it on my Outlook is challenging, just because I have walk*-*in clinics.*

This contrasted with overall sentiments that the intervention required less time and effort than initially anticipated given participant flow as described by another social worker:

$\ldots$*it’s not like we’re seeing this huge amount of patients. But I think that when you’re presenting it to people*$\ldots$*it’d be helpful to kind of inform them of that up front*$\ldots$*but it’s not that big of a workload difference.*

### Design quality and packaging

The providers reported that the intervention packaging provided useful and novel tools for facilitating alcohol-related discussions:

Social Worker 1: *This is more structured, I didn’t have readiness tools and things like that, but, which are great*.

Social Worker 2: *I also like the feedback tool, because we don’t always have like their levels right in front of us, I mean we can obviously click through the whole chart and look at all of their labs*$\ldots$*So it puts it in a nice neat package in terms of providing them with the feedback and having all the normative data and everything else.*

These sentiments were shared by the Psychologists:

Psychologist 1: *One, we sent them home with a sort of a homework form [and we said, bring it back]. I think that has the effect in some ways of sort of holding them accountable, when you have to write down what you’ve done. Write your sins down.*

Psychologist 2: *In my experience, the feedback form was absolutely helpful, and because it allowed them to*—*both of them to, whether they wanted to see how much they were drinking or not, and related it to specific incidents, that allowed them to see those triggers.*

Providers felt that the initial packaging would be improved with more accurate representation of the impact on workflow. As described by one Social Worker:

*I think you can*—*just by acknowledging, “you’re probably already using these, and you’re probably already doing this, we just want to give you a format and kind of keep track of it.” Because you’re not indicating that you’re adding any more work on*$\ldots$*So you’re not adding on anything more on their caseloads. Especially like if you’re doing it within the ID clinic, and the social workers within the clinic are already meeting with these patients. So, I think it’s just acknowledging that.*

In addition to recommendations regarding how the intervention is packaged to the providers, there was a perceived need for additional patient-centered materials as it related to communicating about pharmacotherapy treatment options:

*For some of my patients, not just in the trial, they’ve had prior experience with disulfiram. And they don’t want to go there. And they lump all the medicines together into I’m talking to them about disulfiram. It doesn’t matter what I say, it’s just like the Charlie Brown, waw waw, waw waw, waw waw. They thinking in their mind, disulfiram, if I slip up I’m going to get sick and die, I can’t take medication. It would be nice to have like a table or something to say, “This is this medicine, it’s what it does; this is this medicine, this one’s different”*$\ldots$

## Inner Setting Domain: HIV Clinics

Addressing unhealthy alcohol use was compatible with norms, values, and basic assumptions of the VA, and integrated care was compatible with VA priorities (i.e. creating a medical home) but implementation seemed to be affected by networks and communication, the implementation climate and readiness for implementation.

### Networks and communications

Providers reported that communication among those providers who were co-located in HIV clinics was strong and this facilitated implementation of the intervention. For example, there was fluid and ongoing communication between ID providers and Social Workers:

*And the doctors refer as they’re seeing patients that have the social work-related concerns, as needed.*

Among providers who were not traditionally based in the clinic, however, there was an expressed need for tighter links of communication. As one Addiction Psychiatrist stated:

*One thing I would like is*$\ldots$*a local call*—*a local meeting*—*that would be very helpful too. See what’s going on*$\ldots$*It would be nice to hear what’s going on.*

This was also noted by the Social Workers regarding communication with the Addiction Psychiatrists:

Social Worker 1: *I know we’re all only a phone call away but we don’t*—*also if you have elevators at that level where they’re seeing the addiction psychiatrist, they’re not necessarily having contact with us anymore, so there’s not that communication on that level.*

Social Worker 2: *Oh yeah, we work as a team. I knock on their door, they knock on my door, no, we definitely work as a team, except, the same thing, the addiction psychiatrist is in a separate part of*—*you know, our building is very large.*

### Implementation climate

Aspects (i.e. sub-domains) of the implementation climate most impacting the implementation climate included the tension for change, compatibility, and relative priority.

#### Tension for change

There was a recognized need for integrated addiction services. As described by one of the Addiction Psychiatrists:

*That’s something if a position can be carved out*$\ldots$*and there is a need. There is such a need. Because I get so many phone calls.*

Similarly, developing effective models for treating unhealthy alcohol use were recognized as a priority among VA leadership, with a current focus on specialty settings:

*I would say we’d be supported at the national level*$\ldots$*But right now, frankly our challenge is getting*—*where we’ve been focusing our energy is getting the specialty programs to prescribe medication for alcohol dependence or use disorders. The uptake is really low, nationally.*

#### Compatibility

The Social Worker-based intervention (i.e. the BNI and booster) was highly consistent with ongoing efforts:

*It’s basically what we*—*I mean, at least me, what I have been doing.*

Social Workers who pre-implementation were embedded into the HIV clinics (see Table 1) conduct comprehensive assessments, which typically include an evaluation of alcohol use, as part of routine care promote compatibility of the intervention:

*The way that we operate is, I see all of our new patients ideally like when either they’re newly diagnosed [with HIV] or new to our clinic. Ideally before their initial physician’s appointment, when they come in and get their initial lab work done? I see them, do a comprehensive bio*-*psychosocial assessment, and or provide like post*-*HIV diagnosis counseling, and then see them as needed from there on out, depending on what their resource needs are and psychosocial stressors. And the patients that are long established, I see them either when they come as a walk*-*into see me because they need something, or because the doctors think that they need to see me, or the nurse or the pharmacist or whomever in the clinic.*

The structure was consistent with the PACTs:

*I think it would be a great*$\ldots$*the PACT teams all have now a social worker, a nurse. I think that would be where*$\ldots$*And everybody’s supposed to have a primary care. Should be assigned a primary care. So I think adding this [intervention] in that team, would be*$\ldots$*you would catch the majority of people.*

The intervention was also thought to be consistent with ongoing efforts to promote integrated services:

*So they would be more integrated and they would work more like a team, as opposed to being a group practice of independent providers which is how most mental health clinics are now. And so they’ve been rolling this out for about a year or so, and part of what they’ve talked about is how would you integrate addiction treatment into that.*

#### Relative priority

Addressing unhealthy alcohol use was a priority, particularly when it related to HIV-related outcomes, but one of many priorities:

*But we still, like even before this study I was gonna*—*we used this framework. You know, when you’re dealing with somebody who’s, for example, you go into one of the [clinic] meetings and they’re not taking their HIV meds like they’re supposed to, and they’re developing all this resistance and one of the identified reasons is alcohol, I call them in, talk to them and then hopefully it leads to a referral*$\ldots$*but as our role as social workers to the clinic we’d be neglecting a lot of the other important information if we solely focused on the alcohol without addressing all of the other psychosocial stressors and concerns.*

### Readiness for implementation

Aspects (i.e. sub-domains) most impacting readiness for implementation included available resources and access to knowledge and information.

#### Available resources

In reflecting about potential providers that might be appropriate for delivering the intervention, nurses were not believed to be appropriate:

*We have nurses but our nurses are really directed in nature versus like looking at a framework. This is a lot more probing, and our nurses are much more prescriptive and directive in nature. I don’t know if that’s across the board though.*

#### Access to knowledge and information

There was a perceived need to raise awareness and educate important stakeholders, including patients and HIV providers alike, about pharmacotherapy. As one Addiction Psychiatrist described:

*But something, I mean it makes sense*—*pharmaceutical companies don’t just create something and publish some papers and let it go at that. In order to get people to uptake it they do proactively go out and detail. So we really shouldn’t be surprised if we publish a paper and people don’t uptake it either*$\ldots$*if we could convince the ID docs to at least initiate it, to talk about naltrexone a little bit, and maybe we don’t have to burden them with managing it. But if we could get them to just sell it a little better.*

Other Addiction Psychiatrists also commented on their challenges with initiating pharmacotherapy to treat alcohol use disorders:

Addiction Psychiatrist 1: *One guy came almost every session. Unfortunately he just said, “I like to talk to you, not like to take medicine from you.”*

Addiction Psychiatrist 2: *I have been more successful prescribing SSRI’s and [tricylics rather than acamprosate]. There’s a fair bit of resistance [to pharmacotherapy]*$\ldots$*It’s like, some of*—*a good number of them have been in AA, so you don’t need a pill*—*it’s*-*anything. That resistance, I don’t know*—*I don’t need a pill to*–*I can do it on my own. A lot of patients, it’s from the AA end. And probably some of the pressure is also from… their primary care docs or their cardiologists have never thought about naltrexone.*

## Characteristics of Individuals Domain: Providers (i.e. Social Workers, Psychologists and Addiction Psychiatrists)

Providers endorsed high self-efficacy regarding their implementation, but voiced a need for more consistent utilization of the intervention to maintain skills and discussed alternative models and roles for delivering the components of the intervention.

### Self-efficacy

One Social Worker expressed her enthusiasm for the intervention and comfort with implementing the intervention:

*I’d be seeing them anyways. And so I’m glad to know that you have educated me, and it fits. It definitely fits with what we’re doing.*

This provider attributed treatment success to be most related to the participants and did not attribute it to her treatment or the intervention:

*I think level of engagement has something to do with it. When we talked, I*—*the first client that I had was just so into it that by the time the second session, he was way ahead, way ahead, and so all I could do was affirm what he was doing.*

Low patient volume necessitated relearning of materials. One Psychologist stated:

*And there’s too much time in between [patients]. It’s almost like having to relearn it every [time].*

### Individual stage of change

Whether HIV providers should be responsible for delivering components of the intervention (e.g. the BNI) was questioned by a Social Worker and Addiction Psychiatrist. As described by one Social Worker:

*But if it’s such a brief thing, and they already have a relationship with their physician, doesn’t it make sense to have the physician do this, and then they have the MET with a social worker, I feel like most social workers that I come cross in the VA, could probably do the MET [stuff].*

One Addiction Psychiatrist also agreed that the HIV provider would be effective at delivering pharmacotherapy:

*I think that it could also be powerful if the medication was coming from the Infectious Disease doc, the person who saved my life saying, I think it’s important for your health and your management of your HIV disease, that you stop drinking, and I’m willing to work with you*$\ldots$*I do think that there’s something powerful when the doc who’s saved your life says, “I think you should do something.” That carries a lot of weight. As opposed to this other person I referred you to. So that would be my only suggestion. But I don’t know if you could get ID docs to*$\ldots$*I would submit that they have great motivational skills, because if they can get folks to take HIV meds, then they know how to motivate people.*

## Discussion

Focusing on three CFIR domains, we examined factors impacting the implementation of a model of stepped care for unhealthy alcohol use in HIV clinics. Based on the experiences of Social Workers, Psychologists and Addiction Psychiatrists from five different clinics, several factors emerged as deserving careful consideration for future implementation efforts. First, the providers deemed patients as not typically seeking treatment, and thus materials and processes for facilitating patient motivation for treatment of their unhealthy alcohol use were key and should have greater flexibility. Second, treating unhealthy alcohol use in HIV clinics was perceived by providers to be consistent with VA priorities, which are focused on both screening for and intervening upon unhealthy alcohol use and promoting medical home models of care. Third, depending on the existing role of the provider, there was variability in the perceptions regarding the ideal model for promoting integrated care. These data represent a novel contribution to the literature as they shed light on important factors deserving consideration in implementation of a stepped care model for unhealthy alcohol use in HIV clinics and identify actionable opportunities for future implementation efforts.

Because HIV-infected patients with unhealthy alcohol use were generally not seeking treatment, an advantage of the integrated stepped care model included the tools and process which facilitated patient motivation for treatment. Prior work has demonstrated that HIV-infected patients drink to cope with negative emotions, to promote social interactions, and in response to social pressure [28]; these motivations might be a barrier to treatment. The design of the integrated stepped care model (i.e. Social Worker and Psychologist-delivered manualized counseling focused on enhancing patient motivation to change their alcohol consumption and structured, personalized feedback for patients) facilitated its implementation. While the providers found these techniques to be familiar, they found that the integrated stepped care model provided them with new techniques and materials for enhancing patient motivation, which they perceived to be a barrier to promoting treatment. Indeed, these tools may serve to reinforce motivations to decrease alcohol consumption that have been previously identified as reasons that HIV-infected patients limit their drinking (i.e. concerns about the impact of alcohol use on social functioning, lifestyle incompatibility and physical and mental functioning) [70].

Importantly, treating unhealthy alcohol use through clinic-based settings was perceived by providers to be consistent with VA priorities. Specifically, there have been extensive efforts to promote evidence-based AUDIT-C screening followed by appropriate intervention (i.e. brief intervention, specialty care) for unhealthy alcohol use across VA settings [71, 72]. In contrast, uptake of evidence-based pharmacotherapy for alcohol use disorders has been slow [73–75] despite VA guidelines supporting the use of pharmacotherapy [76]. Addiction Psychiatrists generally recognized accessible pharmacotherapy as an important treatment option for alcohol use disorders, yet reported variable success in initiating treatment. This suggests that patient and HIV provider-targeted education and marketing may be important for improving uptake among non-treatment seeking individuals in the setting of shared-decision making after consideration of all available treatment options [77].

Furthermore, providers found that the integrated stepped care model was consistent with VA priorities given recent initiatives to develop and evaluate new models for delivering screening and treatment for unhealthy alcohol use. Providers also perceived the integrated stepped care model as consistent with PACT as well as PC-MHI and HPDP initiatives, which has important implications for potential future scalability. Such models tend to promote improved communication and tighter networks and allow for flexibility in session duration and specific content, as desired by providers in this study.

While providers were consistent in their reported beliefs that the integrated stepped care model offered a high quality intervention, there were differences based on provider experience and type regarding the ideal way in which to integrate treatment of unhealthy alcohol use into HIV clinics. For example, while some Social Workers believed it was ideal for social workers to deliver the BNI, other suggested that either Infectious Disease Physicians or nurses might provide these services. Additionally, they felt they had the requisite skills and experience to provide the MET intervention. Similarly, it was suggested by Addiction Psychiatrists that Infectious Disease Physicians might be best positioned to engage patients in treatment for unhealthy alcohol use. That a tension exists regarding how to best integrate services is not surprising. On one hand, Infectious Disease and HIV-specialty trained physicians, who increasingly serve as the primary care physician for HIV-infected patients, have an existing relationship with the patient and are managing other treatments. However, prior multi-site studies indicate that HIV providers are often unaware of their patient’s alcohol use and often do not discuss it, even with patients with higher levels of drinking [29, 30, 78]. Moreover, only 65 % of HIV providers reported high comfort with discussing substance use [32]. Thus, it is likely that team-based approaches as structured in the integrated stepped care model, which do not rely on HIV providers to deliver treatment for unhealthy alcohol use, are likely to be an important strategy for effectively addressing unhealthy alcohol use. This model is consistent with others developed to treat other substance use disorders (i.e. buprenorphine for opioid use disorders in HIV clinics) [79–81] and supports the integration of social workers, Psychologists and Addition Psychiatrists into HIV clinics. If effectiveness of integrated stepped care for unhealthy alcohol use in HIV clinics is demonstrated, future studies should focus on determining the optimal role of HIV providers, based on their knowledge and self-efficacy, for delivering such treatments. In addition, the extent to which such an intervention should target other health behaviors, such as drug use or sexual risk behaviors, warrants investigation.

 The results of this study should be interpreted in the context of its limitations. First, the providers represented a convenience sample of individuals involved with an ongoing randomized controlled trial. Our results may not reflect the opinions of other providers involved with the randomized controlled trial or apply to other VA-based providers more generally. Second, our interview guides were not piloted in advance of our study. In retrospect, given the expressed need to address alcohol along with other drug use and HIV prevention, we would have asked Addiction Psychiatrists further about their comfort prescribing pharmacotherapy for other substances (i.e. tobacco, opioids, cocaine) using an integrated model and how they might address HIV risk behaviors and prevention. Third, these findings may not transfer universally to other HIV clinical settings, particularly settings where Psychologists and Addiction Psychiatrists are less available. The goal of the *STEP Trials* is to evaluate the effectiveness of integrated care from Psychologist and Addiction Psychiatrists, along with Social Workers. If effectiveness is demonstrated, issues of generalizability of this and the main study’s findings to other HIV clinical settings will be carefully considered. Fourth, this qualitative study was conducted during the 1st year of implementation of integrated stepped care in the context of a randomized controlled trial. Whether these findings will apply at later stages is unclear; though this study does provide important insights to inform planning phases and initial implementation of integrated stepped care models. Fifth, given our small sample size, we are unable to determine whether we had reached thematic saturation. Regardless, this study provides meaningful data given its focus on implementation of a novel model of care for unhealthy alcohol use in HIV clinics based on a robust deductive approach to data analysis, which revealed consistency in findings within and across different groups of providers. Sixth, our study may have been subject to social desirability bias given the proportion of interviewers relative to interventionists; however, we felt that the diverse perspectives of the interviewers served to enhance the richness of the conversation and data collection.

## Conclusions

In conclusion, our study represents an important step in identifying key considerations based upon the CFIR constructs when implementing an integrated stepped care model for unhealthy alcohol use in HIV clinics. We found that implementation of this model may be facilitated by tools to help providers enhance patient motivation, close alignment with organizational values and existing models of care, and optimization of provider self-efficacy and roles. Future efforts aimed at obtaining the perspectives of additional providers, as well as patients and HIV providers will be important for developing a comprehensive understanding of factors impacting implementation of integrated stepped care for unhealthy alcohol use in HIV clinics. In addition, consideration of alternative models involving various providers completing the primary components of the intervention, will be important for informing the development of successful interventions.
